# Evidence of the shifting baseline syndrome in ethnobotanical research

**DOI:** 10.1186/1746-4269-9-75

**Published:** 2013-11-14

**Authors:** Natalia Hanazaki, Dannieli Firme Herbst, Mel Simionato Marques, Ina Vandebroek

**Affiliations:** 1 Laboratory of Human Ecology and Ethnobotany, Ecology and Zoology Department, Federal University of Santa Catarina, ECZ-CCB-UFSC, Florianópolis, SC 88010-970, Brazil; 2 Post Graduation Program in Ecology, Federal University of Santa Catarina, Florianópolis, Brazil; 3 Post Graduation Program in Plant Biology, Federal University of Santa Catarina, Florianópolis, Brazil; 4 Institute of Economic Botany, The New York Botanical Garden, 2900 Southern Boulevard, Bronx, NY 10458, USA

**Keywords:** Traditional ecological knowledge, Ethnoecology, Intra-cultural variation, Environmental perception

## Abstract

**Background:**

The shifting baseline syndrome is a concept from ecology that can be analyzed in the context of ethnobotanical research. Evidence of shifting baseline syndrome can be found in studies dealing with intracultural variation of knowledge, when knowledge from different generations is compared and combined with information about changes in the environment and/or natural resources.

**Methods:**

We reviewed 84 studies published between 1993 and 2012 that made comparisons of ethnobotanical knowledge according to different age classes. After analyzing these studies for evidence of the shifting baseline syndrome (lower knowledge levels in younger generations and mention of declining abundance of local natural resources), we searched within these studies for the use of the expressions “cultural erosion”, “loss of knowledge”, or “acculturation”.

**Results:**

The studies focused on different groups of plants (e.g. medicinal plants, foods, plants used for general purposes, or the uses of specific important species). More than half of all 84 studies (57%) mentioned a concern towards cultural erosion or knowledge loss; 54% of the studies showed evidence of the shifting baseline syndrome; and 37% of the studies did not provide any evidence of shifting baselines (intergenerational knowledge differences but no information available about the abundance of natural resources).

**Discussion and conclusions:**

The general perception of knowledge loss among young people when comparing ethnobotanical repertoires among different age groups should be analyzed with caution. Changes in the landscape or in the abundance of plant resources may be associated with changes in ethnobotanical repertoires held by people of different age groups. Also, the relationship between the availability of resources and current plant use practices rely on a complexity of factors. Fluctuations in these variables can cause changes in the reference (baseline) of different generations and consequently be responsible for differences in intergenerational knowledge. Unraveling the complexity of changes in local knowledge systems in relation to environmental changes will allow the identification of more meaningful information for resource conservation.

## Background

Traditional ecological knowledge (TEK) is an important component in the improvement of natural resource management [1-4] and in the practices related to protection of ecosystems and species [5]. This kind of knowledge is developed by local communities through adaptive experiences with natural resources. It is dynamic and continuously modified, yet generally little emphasis is given to understanding changes as adaptive responses to new environmental, social, and economic conditions [6]. Such changes can also be related to a “loss of knowledge”, especially when the social reproduction of people holding TEK is at risk, resulting in the loss of local knowledge systems. The loss of local knowledge can result in a diminished ability to cope with environmental alterations, and also can be related to a changing baseline in the perception of natural resources.

These different references in relation to a baseline can be understood under the  *shifting baselines syndrome’*, proposed by Pauly [7] in a seminal paper describing the reasons and implications of a syndrome occurring among fishery scientists. Pauly [7] noticed that each generation of scientists considers as a baseline the abundance and composition of species observed at the beginning of their careers, and use this baseline to evaluate changes along time. Following the discussion of this syndrome some authors argue that a similar trend may be occurring among fishermen [8-12], in studies about forest cover changes [13,14], bird fauna, and agriculture [15].

This syndrome allows a historical approach in assessing an environment, which can be combined with aspects of the current local situation [16]. For a study on shifting baseline it is necessary to analyze information on processes of change in the environment, resources, or any other conditions, using the perception of the people who observe or follow this process [4].

One of the problems is that, for several areas and species, there are no well-known starting points, or baselines [16]. This indicates a potential weakness in studies where researchers may not be comparing the environment (or a resource) from an earlier baseline, since reference points are considered dynamic. Combining data from different sources may be the only way to derive trends on the shifting baseline syndrome, when no consistent historical data is avaliable [17].

Ethnobotanical research addressing the shifting baseline on vegetation or plant resources is still very scarce and recent [13,14]. Similar to changes in fish stocks observed under the shifting baseline perspective, vegetation and forest cover change over time; along with it people’s perceptions about plant species and landscapes are also subject to change, yet those changes may remain unnoticed or underperceived by different generations. Several factors resulting from socioeconomic changes influence landscape alterations and the use and availability of plant resources, such as monoculture farming, real estate speculation, tourism, and urbanization, among others. As changes occur in social, economic and environmental conditions at a given location, it is expected that local people’s knowledge also changes between different generations [18]. These changes can be accompanied by a gradual accommodation of people’s perceptions, with which the dynamics of reference points are directly related. If changes in vegetation, terrestrial landscapes and the co-ocurrence of people’s accommodations of these changes (for example through studies of plant knowledge across different generations) were analyzed together, then there would be a better understanding of people’s tolerance in relation to biodiversity loss.

The main objective of this paper is to analyze the ideas behind the shifting baseline in the context of ethnobotanical research. Evidence of the shifting baseline syndrome can be found in studies dealing with intracultural variation of knowledge, when knowledge from different informant generations is compared and changes in the environment and/or resources are also mentioned. In other words, we are assuming that there is evidence of shifting baseline in a study when: (1) research data in the paper point to differences in intergenerational knowledge, with knowledge being lower in younger generations; (2) local community members or the researchers themselves mention that one or more biological resources are disappearing. The latter information was most often encountered in the papers as anecdotal evidence. Our main argument is to reinforce the role of this evidence of environmental change when analyzing age differences found in studies dealing with the distribution of ethnobotanical knowledge in a given group. A review of ethnobotanical studies was conducted, which investigated age differences and changes in local knowledge. The intent is to add more elements to the analysis of traditional knowledge in ethnobotany, where traditions and transformations are intrinsically mixed.

## Methods

We used the bibliographic databases Scopus, Biological Abstracts and Medline for this review, covering studies published between 1993 and 2012. The goal was to select studies with comparisons of ethnobotanical knowledge according to different classes of age or age groups, in order to make inferences regarding changes in baselines interfering in people’s perceptions. Thus, the variable “age” was a priority for the selection of studies, recognizing that there are other important variables influencing the ethnobotanical repertoire. In this search the keyword “ethnobotany” was used added to a combination of expressions: “shifting baseline”, “age”, “age comparisons”, “older” and “younger”, “age class”, “age group”, “knowledge”, “knowledge loss”. A total of 168 studies were found, of which 84 were selected for analysis, according to two inclusion criteria: the studies had to involve age group comparisons of ethnobotanical knowledge and to show results on, or discuss changes in, the abundance of local plant resources. Excluded were studies, for example, that focused on the ethnopharmacology of a given plant without information about age comparisons.

Analysis consisted of ranking the papers from the literature search into four categories: (1) evidence of shifting baseline; (2) no evidence of shifting baseline; (3) no changes in knowledge occur; and (4) ambiguous. We considered “evidence of shifting baseline” when a paper showed differences in intergenerational knowledge (lower knowledge levels in younger generations) and mentioned declining abundance of natural resources (through own research or from the literature). “No evidence of shifting baseline” occured when there were intergenerational differences in knowledge but there was no information in the paper about the environment or abundance of natural resources. “No changes” was when there were no intergenerational differences, usually when knowledge was widely shared, independent of perceived changes in the environment. Some studies were not clear about evidence of declining of resources or changes in the environment and were considered “ambiguous”.

After analyzing the studies for evidence of the shifting baseline syndrome, we also searched within these studies for the use of the expressions “cultural erosion”, “loss of knowledge”, or “acculturation”. These concepts are often used to explain differences in knowledge and perceptions occurring in different age classes. We believe that these concepts should be treated with a lot of caution, since one cannot conclude straightforward that there is a process of cultural erosion, acculturation, or loss of knowledge when simply making comparisons between observed knowledge in different age groups. First of all, learning and experiences require time. Therefore, an alternative explanation is that older people tend to accumulate knowledge over time compared to younger people. Second, older people have different perceptions than younger people because their reference points are different.

## Results

The 84 selected studies (Table 1) comprised ethnobotanical research from different parts of the world, predominantly Brazil (17 studies) and Ethiopia (13 studies), followed by Argentina, Burkina Faso, and Mexico (5 studies each), India and Peru (3 studies each), and lastly Benin, Italy, Kenya, Micronesia, Phillipines, Spain, Thailand, Turkey and Uganda (2 studies each). Fifteen studies were conducted in other countries. Some of these studies included more than one paper published from the same original dataset (*e.g*. [19-22]), and in this case we analyzed their results as a group to avoid pseudo-replication.

**Table 1:** Studies analyzed, studied region, and type of resource

**Reference**	**Studied region**	**Resource**
Albino-García *et al*. [23]	Puebla, Mexico	weeds
Albuquerque *et al*. [24]	Northeastern Brazil	medicinal plants
Almeida *et al*. [25]	Northeastern Brazil	medicinal plants
Almeida *et al*. [26]	Northeastern Brazil	medicinal plants
Awas *et al*. [27]	Blue Nile, Ethiopia	useful plants
Ayantunde *et al*. [28]	Southwestern Niger	herbaceous and woody plants
Badshah and Hussain [29]	Pakistan	medicinal plants
Balslev *et al*. [30]	Peruvian Amazon	uses of one species
Bognounou *et al*. [31]	Burkina Faso	uses of five species
Brosi *et al*. [32]	Pohnpei, Micronesia	plants used for canoe building
Caniago and Siebert [33]	West Kalimantan, Indonesia	medicinal plants
Carbajal-Esquivel [34]	San Luis Potosí, Mexico	food plants
Case *et al*. [35]	Papua New Guinea	useful plants, with medicinal emphasis
Cilia-Lopez *et al*. [36]	San Luis Potosí, Guanajuato, Querétaro, Mexico	uses of one species
Cruz-García [37]	Western Ghats, India	wild food plants
De Beer and Van Wyk [38]	Northern Cape Province, Southern Africa	useful plants
De Caluwé *et al*. [39]	Northern Benin	uses of one species
Della *et al*. [40]	Cyprus	wild food plants
Eilu *et al*. [41]	Tororo, Uganda	indigenous plants
Esser *et al*. [42]	Ethiopia	uses of one species
Estomba *et al*. [43]	Patagonia, Argentina	medicinal plants
Estrada-Castillón *et al*. [44]	Sierra Madre Oriental, Mexico	medicinal plants
Figueiredo *et al*. [45]	Sepetiba Bay, Brazil	medicinal plants
Flatie *et al*. [46]	Assosa Zone, Ethiopia	medicinal plants
Franco and Barros [47]	North/Northeastern Brazil	medicinal plants
Ghorbani *et al*. [48]	Yunnan, China	wild food plant
Giday *et al.*[49]	Southwest Ethiopia	medicinal plants
Giday *et al.*[50]	Meinit-Goldya, Ethiopia	medicinal plants
Giday *et al.*[51]	Southwest Ethiopia	medicinal plants
González *et al.*[19-22]	Spain	medicinal, cosmetic, repellent and edible plants
Hanazaki *et al.*[52]	Southeast Brazil	useful plants
Houessou *et al.*[53]	Benin	uses of one species
Idolo *et al.*[54]	Italian Apennines	useful plants
Karunamoorthi and Husen [55]	Oromia, Ethiopia	repellent plants
Karunamoorthi *et al*. [56]	Ethiopia	repellent plants
Karunamoorthi *et al*. [57]	Kofe Kebele, Ethiopia	repellent plants
Kristensen and Balslev [58]	Nazinga Game Ranch, Burkina Faso	woody plants
Kristensen and Lykke [59]	Burkina Faso	woody plants
Lacuna-Richman [60]	Leyte Island, Philippines	non-timber forest resources
Ladio [61]	Patagonia, Argentina	wild edible plants
Ladio and Lozada [62]	Patagonia, Argentina	wild edible plants
Lee *et al*. [63]	Micronesia	food plants, plants for fish poison and canoes
Lima *et al*. [64]	Central Brazil	native trees
Lins Neto *et al*. [65]	Northeast of Brazil	uses of one species
Luziatelli *et al*. [66]	Junín, Peru	medicinal plants
Lykke *et al*. [67]	Burkina Faso	woody plants
Lyon and Hardesty [68]	Madagascar	medicinal plants
Martínez and Lujan [69]	Central Argentina	veterinary plants
Matavele and Habib [70]	Mozambique	medicinal plants
Mathez-Stiefel *et al*. [71]	Bolivia and Peru	medicinal plants
Mcmillen [72]	Tanga, Tanzania	medicinal plants
Merétika *et al*. [73]	Southern Brazil	medicinal plants
Miranda *et al*. [74]	Southeast Brazil	useful plants
Monteiro *et al*. [75]	Northeastern Brazil	uses of two species
Olowa *et al*. [76]	Phillipines	medicinal plants
Panghal *et al*. [77]	India	medicinal plants
Phillips and Gentry [78]	Madre de Dios, Peru	useful plants
Polo *et al*. [79]	Spain	uses of one species
Quave *et al*. [80]	Southern Italy	medicinal plants for dermatological problems
Quinlan and Quinlan [81]	Dominica	medicinal plants
Ramos *et al*. [82]	Northeastern Brazil	fuelwood
Rana *et al*. [83]	India	wild edible plants
Sarper *et al*. [84]	Turkey	wild plants
Schunko *et al*. [85]	Austria	wild plants
Seid and Tsegay [86]	South Wollo, Ethiopia	medicinal plants
Silva and Proença [87]	Central Brazil	medicinal plants
Silva *et al*. [88]	Northeastern Brazil	medicinal plants
Silva *et al*. [89]	Northern Brazil	fruits and plants
Simsek *et al*. [90]	Turkey	wild plants
Smith-Oka [91]	Veracruz, Mexico	medicinal plants
Sop *et al*. [92]	Burkina Faso	woody plants
Srithi *et al*. [93]	Thailand	medicinal plants
Srithi *et al*. [94]	Northern Thailand	medicinal plants
Stave *et al*. [95]	Turkana, Kenya	woody plants
Tabuti *et al*. [96]	Uganda	medicinal plants
Tanaka *et al*. [97]	United States	medicinal plants
Teklehaymanot [98]	Dek Island, Ethiopia	medicinal plants
Terer *et al*. [99]	Kenya	uses of one species
Toledo *et al*. [100]	Central Argentina	medicinal plants
Uprety *et al*. [101]	Nepal	wild edible plants
Voeks and Leony [102]	Northeastern Brazil	medicinal plants
Yineger and Yewhalaw [103]	Ethiopia	medicinal plants
Yineger *et al*. [104]	Jimma Zone, Ethiopia	medicinal plants
Zuchiwschi *et al*. [105]	Southern Brazil	woody plants

**Figure 1:**
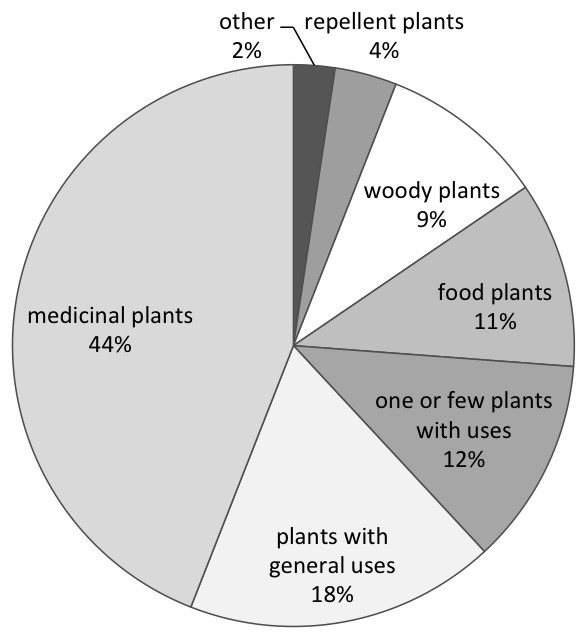
**Types of plant resources investigated in 84 studies on ethnobotany and age comparisons (“other” includes plants with veterinary purposes and plants used to build canoes).** Values in percentage.

The keywords used in the search resulted in a compilation of studies with different goals. Nonetheless, all studies analyzed ethnobotanical knowledge according to the age of informants. Articles were grouped by their similarity of plant uses (*e.g*. medicinal plants, food plants, Table 1). The studies also differed in level of detail when defining the resource type analyzed. Forty four percent of studies focused on medicinal plants (Figure 1), which included detailed uses such as “medicinal plants for dermatological problems” [80] and studies that concerned the generic grouping of medicinal plants (*e.g*. [66]). The category “general uses” included studies investigating a set of useful plants (*e.g*. [38]), herbaceous and woody plants for general uses (*e.g*. [28]), and indigenous plants (*e.g*. [41]). Some studies investigated the uses of one (*e.g*. [79]) or a few species (*e.g*. [31]) and were included since the choice to investigate their ethnobotany is already biased by the local importance of those species. Studies of food plants included studies of edible wild plants (*e.g*. [83]) and food plants in general, including fruits (*e.g*. [89]).

**Figure 2:**
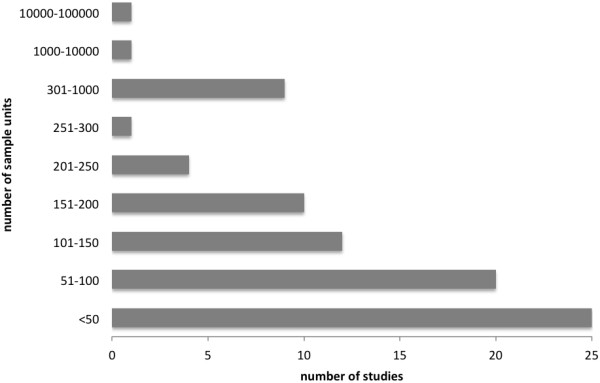
Number of subjects or interviews (sample units) from the 84 studies on ethnobotany and age comparisons.

Sampling methods and data collection varied according to the objectives of each study. Data collection through interviews included both intentional sampling, and systematic sampling, the latter being a sampling procedure with a higher degree of randomness. Other data collection tools included focus group discussions and participative workshops. Sample sizes were highly variable (Figure 2), ranging from 13 subjects [104] to more than 90,000 subjects [97]. There was also diversity among ecosystems and human groups studied, as well as types of data analyzed. For example, although most studies focused on the knowledge about plant resources, there were studies dealing with knowledge associated with the broad use of a given resource, such as in Brosi *et al*. [32] who studied knowledge of canoe building as a whole.

It is important to consider that the comparison between age classes is relative, not absolute. Therefore, age classes vary among the studies. In some studies they were set at intervals of approximately 5 years [24,57] or 10 years [35,88]. Other studies separated the informants into two age groups, usually 40 years of age being the boundary between the groups [45,49-52,65,70,89], although this boundary varied depending on the location studied. For example, Flatie *et al*. [46] split informants into two age groups: those with more than 15 years of age and those younger than 15 years. In other studies comparisons were done without creating specific age classes [31,59,76,82,83,95,100,104]. Lastly, some studies did not clearly define how the separation between age groups was done [30,37,40,41,47,93].

Over half of the 84 studies discussed a concern towards cultural erosion or knowledge loss (57%), using these arguments to explain the results found. These arguments were absent in 43% of the studies. Sometimes these expressions were used with a more detailed discussion of occurring changes, such as Quinlan and Quinlan [81] who considered the subtle and complex effects of modernization on traditional medicine. Other authors mentioned cultural erosion or loss of knowledge, but considered that these phenomena would not occur in their case because ethnobotanical knowledge was widely shared (e.g. [59,95]). We also need to keep in mind that the dominant epistemological paradigms to explain observed phenomena can change over time, and the concept of “cultural erosion” could become replaced by more recent ideas linked to adaptability and environmental change.

**Figure 3:**
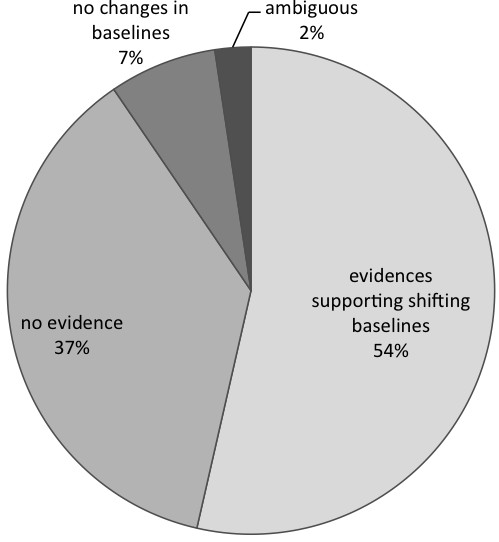
**Possible evidence of shifting baselines syndrome from 84 studies on ethnobotany and age comparisons.** Evidence supporting shifting baselines occur when there are age differences in combination with environmental changes reported by informants or the authors (see Methods for further details).

More than half of the 84 articles (54%) showed some evidence of the shifting baseline syndrome, through the existence of intergenerational differences in knowledge and information about the declining of biological resources reported by local community members or by the researchers. Usually the researchers made that observation anecdotally, either from reports by participants, or from their own observation of the local situation (Figure 3). Some of these evidences were subtle, such as in Eastern Uganda where “some indigenous plants were reported to have disappeared or become scarce” [41]. Similarly, in southern Madagascar, traditional medicine might not be threatened by the loss of primary forest, because people can turn to exotic plants from disturbed locations [68]. About 37% of the studies did not provide any evidence of shifting baselines, generally by not taking into account any reported or literature information about the environment or the abundance status of plant resources, but also because they did not show any intergenerational differences about the decline of resources reported or any knowledge changes. In 7% of the studies there were no intergenerational differences and knowledge was widely shared, and this could be independent of perceived changes in the environment. Interestingly, these studies were predominantly those that investigated the use of one or a few species [30,39,42], which tended to be selected for research precisely because they were culturally important species. In a small percentage of articles, the evidence appeared to be ambiguous. For example, in a preserved region studied by Idolo *et al*. [54], none of the species with reported past uses had gone extinct in the area, but less than a quarter of uses previously recorded were still present in people’s life, showing that the resources are likely to be available, but few of them currently in use. Thus, we could not clearly infer if there was evidence of shifting baseline in this case.

## Discussion and conclusions

This article illustrates the complexity of perspectives on plant knowledge at different ages. Declining knowledge due to disruptions in the social transmission of knowledge between generations has been widely reported in ethnobotanical studies (*eg*. [29,40,51,80], in several cases to a worrisome degree. The results highlighted in this paper show that, in addition, it is necessary to pay concurrent attention to the status of environmental changes that may reflect declining plant resources. Such ecological changes that contribute to the loss or declining availability of plants obviously can also lead to the loss of valuable information within traditional knowledge systems [27], and this is mediated by changes in people’s perceptions about these resources.

Knowledge variation over generations has been explained in different ways. Some authors associate intergenerational variations, in ethnobotanical repertoires, with loss of knowledge [35,52], acculturation [106], or modernization [81]. For resources such as medicinal plants, it is argued that there exists a trend in which this knowledge is acquired over the life of each individual, and accumulated in older age groups as compared to younger people [76,78]. Therefore, considering that more than one third of all studies analyzed in this paper focused on medicinal plants, age was identified as a major factor influencing ethnobotanical knowledge [45,68,86,93,104]. A counterpoint is provided by Luziatelli *et al*. [66], who considered that although there is a general trend towards acquiring medicinal plant knowledge throughout age, much of the variation between informants can be explained by personal interests and also by their relationship with a local healer, demonstrating the individual influence in knowledge transmission and maintenance. In another study, young healers who had many practicing family members had a similar amount of plant knowledge as older, more experienced healers with a smaller social entourage [107]. The authors concluded that the social component of medicinal plant knowledge may explain these results. Strong family ties enable young healers to assimilate knowledge about medicinal plants rapidly from experienced relatives, while older healers with few practicing family members but many years of experience with medicinal plants also had high knowledge scores.

Another explanation for higher medicinal plant knowledge among older people is the lack of interest from youth regarding these resources and associated practices [26,47,50,73,88,104], as well as the type of health services predominating among younger people and their accessibility [45,70,73,81], and changes in lifestyle and the environment in terms of availability of plant resources [86]. The argument here is that decreasing knowledge and declining plant resources can be phenomena that are occurring together. It is not the goal here to find out which came first, but to acknowledge their combined effects and to recognize possible long term consequences in shifting environmental baselines regarding plant resources. Additionally, deforestation and lack of access to traditional resources (such as harvesting prohibitions due to environmental regulations) can both affect traditional knowledge of medicinal plants, making “erosion of knowledge” a complex process [24,90], which can be reinterpreted according to new theoretical perspectives and insights in scientific discourse that emerge over time. Conclusions about cultural erosion need to take into account local community voices. Community members compare the current environment and species composition with preterit situations experienced, and thus can be better actors than scientists to draw conclusions about cultural erosion.

It is a limitation to use the number of species cited by each informant as the main (or only) variable to evaluate people’s knowledge because knowledge may be transformed [81,108], although it allows general comparisons. Perhaps it is not enough to analyze the dynamics of knowledge as a whole and to conclude that knowledge is being lost. We also need to consider the limitations of using plant names as a proxy for plant knowledge, since plant knowledge as a whole goes beyond the naming of plants. Also, plant names are not perfect correlated to the number of plant species, owing to under-differentiation (one local plant name refers to different botanical species) or over-differentiation (one botanical species is known by different local names).

It may be necessary to associate the number of plants recognized to the type of use, because while there is a decrease of knowledge for a category of plant (*eg.* medicinal plants), the knowledge can remain stable or increase compared to other categories of use [6]. Also, new plants may be added to ethnobotanical repertoires. Furthermore, to understand the dynamics of knowledge and complexity of the process of “loss” (or rather, transformation), researchers must analyze the changes that occurred in the context where this knowledge exists over time, as well as its causes. Gómez-Baggethun and Reyes-García [6] consider that few researchers are trying to understand how the causes of loss of knowledge affect the mechanisms that allow the societies to generate, regenerate, transmit and apply this knowledge.

In studies focused on food plants, there seems to be less evidence of knowledge misfits between different age groups, namely, the shifting baseline syndrome may not be occurring. This may happen because the contact and experience with those types of plant resources tend to be more evenly distributed within the population, even when one assumes knowledge to be patterned according to variables such as gender, social status, occupation and age itself. People usually have extensive contact with and depend on food plants since their childhood, and people usually experiment with them more often than with medicinal plants [78]. In addition, there is the secrecy aspect of medicinal plants in some human groups who recognize key individuals such as healers or herbalists (*e.g*. [107,109]). In the case of studies about only one or a few species, these species tend to be widely used or have a widespread importance among the communities studied; therefore these studies do not provide evidence of changes in baseline.

Our main point of contention is that the general perception of knowledge loss among young people when comparing ethnobotanical repertoires among different age groups should be analyzed with caution. Almeida *et al*. [25] argued that this information was often used to infer incorrectly the relationship between acculturation and lack of knowledge. More attention should be given to the complexity of these changes. A comparison of knowledge about medicinal plants among Dominicans in rural and urban areas of the Dominican Republic and those who have moved to New York City showed that knowledge of food medicines was not affected by age, whereas younger people had less knowledge of nonfood medicines [108]. This indicated that ethnobotanical knowledge is still alive even in globalized contexts, challenging the paradigm of loss of knowledge about plants [108].

Sometimes medicinal plant knowledge does not depend only on the level of plant diversity, degree of modernization or absence of Western health care infrastructure; other social factors such as the healing tradition of the extensive family, can be also fundamental to the survival of medicinal plant knowledge [107]. Thus, a careful understanding of these complex transformation processes is needed [71]. This also includes an analysis of how the environment has changed over time and how these changes have affected plant resources as well as perceptions about these plant resources.

Baseline changes can be related to different issues that are sometimes linked. First, changes in the landscape or in the abundance of plant resources may be associated with changes in ethnobotanical repertoires held by people of different age groups. According to Sáenz-Arroyo *et al*. [8], there are some species of fish that may have been abundant in certain areas in the past, but currently exist only in historical documents and in the memory of some fishermen and researchers. The same type of phenomena can be observed in ethnobotanical research, in situations where some indigenous plants were reported to have disappeared or become scarce, due to natural causes (such as drought) and/or anthropogenic causes (such as uncontrolled harvesting, clearing for cultivation, firewood extraction, among others) [41]. These losses can be reflected in the ethnobotanical repertoires of local people. Even though our analysis was focused on age differences, we do not discard the role of other variables in this scenario, including changes in gender composition over time, or changes in other important variables such as education or main economic activites.

Second, the relationship between the availability of resources and the current practices of using plants rely on a complexity of factors. Changes in plant species composition over time may result from socio-cultural and economic changes affecting a given human group. Such changes can cause changes in the reference (baseline) of different generations and consequently resulting in a framework of different intergenerational knowledge. According to Baum and Meyers [110], information and knowledge of native species’ diversity and abundance from the recent past is not being transmitted to younger generations. This may be due to shifting patterns of communication between age groups (generations) or because particular resources may no longer be available or of interest. Some resources can come into disuse due to industrialization or technological facilities (such as firewood displaced by gas stoves or medicinal plants displaced by modern medicine). In another study, Rana *et al*. [83] considered several causes for knowledge loss, such as the association of wild food plants with low income. On the other hand, when comparing knowledge of mothers and children, Cruz-García [37] argued that all mothers used to consume more wild food plants before, and reported decreases in collection of these resources due to the decreasing availability of plants rather than to increasing social stigma.

Culture and knowledge are dynamic components in people’s lives, as well as the environment in which they live. According to Brosi *et al*. [32] people often change their techniques when easier methods become available, as part of a gradual cultural evolution. When investigating people’s knowledge about the environment and resources, as well as the dynamics of their knowledge and practices, both changes in the environment and in local livelihoods over time should be considered. Reports based on memories about the past situation of plant resources can be biased by the gradual acceptance of a new baseline [15]. Thus, complementary methodologies in studying human perception and knowledge are needed to reduce biases and assumptions that may arise from local ecological knowledge [17].

Despite the need to collect data that indicate changes in knowledge over time, as well as its causes, we have to assume that there exists a limitation of the human perception about changes in vegetation, because the knowledge about the environment’s past may not show an original condition. In other words, some changes that occurred over time may not be recorded or remembered by individuals and not be known by science [16]. Without historical knowledge about the environment or about a given species, the baseline will continue to change and the risk is gradual acceptance of increasingly lost of rare species [7,8,110]. It is essential to use an interdisciplinary approach, based on a wide variety of data to estimate historical changes and to understand the current changes in a social and historical context [11], since complementary data may support and provide reliability to informant’s reports. In the case of fish stocks and tree resources, older popular literature can be accessed, as well as naturalistic observations, photographs, ancient accounts [8], logbooks [17], monitoring of fish landings [7,17], maps, and other historical data [14].

In the case of plant resources, historical data, old photos, aerial photos, satellite images, and other records of different times may detect changes in vegetation and support data on people’s perceptions of a particular site or resource. Methodologically, the most interesting would be the integration of different methods of collecting and analyzing data, in order to better understand the changes occurring over time and the origin of these changes. According to Godoy *et al.*[111] and Quinlan and Quinlan [81], we still have to face the problems derived from a lack of a reliable baselines to estimate changes in traditional knowledge, which can be partially solved through longitudinal studies replicating the same study in a given place after a time span [81]. Furthermore, comparative ethnobotanical studies spanning multiple generations become increasingly possible due to methodological standardization, which has occurred in the past two decades. Once the extent of the environmental changes and their causes are known, it becomes possible to better understand the changes in knowledge of different generations. Thus, more elements may be added to the simplistic argument of “acculturation” or “loss of knowledge”. Ethnobotany and other areas such as historical ecology can contribute to understanding the changes in reference points through critical analysis of intracultural variations in the perception of local stakeholders, involving both plant species and resources, as well as the landscapes that include them.

## Competing interests

The authors declare that they have no competing interests.

## Authors’ contributions

NH, DFH and MSM, conceptual idea, literature review, data analysis, discussion and writing; IV, literature review, discussion and writing. All authors read and approved the final manuscript.
